# Food security for infants and young children: an opportunity for breastfeeding policy?

**DOI:** 10.1186/s13006-015-0029-6

**Published:** 2015-02-23

**Authors:** Libby Salmon

**Affiliations:** Australian Centre for Economic Research on Health, Research School of Population Health, The Australian National University, Building #62, Corner of Mills & Eggleston Roads, Canberra, ACT 0200 Australia

**Keywords:** Food security, Breastfeeding, Breast milk, Breast milk substitutes, Infant, Nutrition, Trade, Policy, Markets, Regulation

## Abstract

**Background:**

Increased global demand for imported breast milk substitutes (infant formula, follow-on formula and toddler milks) in Asia, particularly China, and food safety recalls have led to shortages of these products in high income countries. At the same time, commodification and trade of expressed breast milk have fuelled debate about its regulation, cost and distribution. In many economies suboptimal rates of breastfeeding continue to be perpetuated, at least partially, because of a failure to recognise the time, labour and opportunity costs of breast milk production. To date, these issues have not figured prominently in discussions of food security. Policy responses have been piecemeal and reveal conflicts between promotion and protection of breastfeeding and a deregulated trade environment that facilitates the marketing and consumption of breast milk substitutes.

**Discussion:**

The elements of food security are the availability, accessibility, utilization and stability of supply of nutritionally appropriate and acceptable quantities of food. These concepts have been applied to food sources for infants and young children: breastfeeding, shared breast milk and breast milk substitutes, in accordance with World Health Organization (WHO)/United Nations Children’s Fund (UNICEF) guidelines on infant feeding. A preliminary analysis indicates that a food security framework may be used to respond appropriately to the human rights, ethical, economic and environmental sustainability issues that affect the supply and affordability of different infant foods.

**Summary:**

Food security for infants and young children is not possible without high rates of breastfeeding. Existing international and national instruments to protect, promote and support breastfeeding have not been implemented on a wide scale globally. These instruments need review to take into account the emerging trade environment that includes use of the internet, breast milk markets and globalised supply chains for breast milk substitutes. New approaches are required to handle the long-standing policy conflicts that surround infant and young child feeding. Placing breastfeeding in a food security framework may achieve the political attention and policy co-ordination required to accelerate breastfeeding rates in a range of economies.

## Background

The World Health Organization (WHO)/United Nations Children’s Fund (UNICEF) Global Strategy for Infant and Young Child Feeding prioritizes exclusive breastfeeding for six months and continuation for up to two years of age or beyond [1]. For the few health situations where this is not possible, and depending on individual circumstances, the strategy recommends the following alternatives: ‘expressed breast milk from an infant’s own mother, breast milk from a healthy wet-nurse or a human-milk bank, or a breast-milk substitute’ ([1] p. 10). Recent developments in the availability of these alternatives and their implications for breastfeeding require examination.

Despite low rates of exclusive breastfeeding globally [2] high-income countries like Australia consider infants to be food secure [3]. However in 2013, countries that export dairy-based infant formula, including Australia, New Zealand, the United Kingdom and Germany were forced to tighten export regulations to maintain domestic supplies and retailers limited the number of tins of infant formula that could be sold to a customer [4-6]. These actions were taken in response to domestic shortages as stock was bought up for private export to Asia, often facilitated by the internet [7]. Unlawful exports of infant formula from New Zealand, largely to China, were worth NZ$150 million in 2013 [4].

Demand by China for imported breast milk substitutes increased rapidly from 2008 when melamine contamination of infant formula manufactured in that country caused the death of six babies and illness in over 300,000 [8]. This ongoing food safety crisis was exacerbated by recalls in 2013 of infant food ingredients from New Zealand [9], which led to disrupted supply chains for infant foods manufactured in Australia and other countries [10].

Looking beyond food safety, the interdependence of supply chains for infant food manufacturing and the scale of China’s demand for imported infant formula [11] represent a crisis in the secure supply of appropriate, affordable infant food – a result of low breastfeeding rates in both China and countries like Australia [12,13]. Demand for breast milk substitutes is also increased by industry cross marketing of infant formula with follow-up formulas and toddler milks, which undermines exclusive breastfeeding to six months and continued breastfeeding thereafter [14,15].

These problems illustrate the globalization of trade in breast milk substitutes and the exposure of infants and young children to food security issues across low- to high-income countries. While trade is the conventional solution to the failure of local supplies of food, it can contribute to food scarcity and make food less affordable elsewhere. Unrestricted trade in infant foods is, in principle, limited by international agreement to protect breastfeeding through the 1981 WHO International Code of Marketing of Breast-milk Substitutes (the WHO Code) [16]. When first adopted, the WHO Code received widespread support but few countries have since implemented it fully [17], or observed equally binding resolutions of the World Health Assembly to include follow-up formula and toddler milks in its scope [14,18].

Another example of an emerging food security issue is the supply and safety of expressed breast milk. Demand for expressed breast milk is driven by a diverse range of factors: an expanding number of breast milk banks for premature and sick infants; mothers who want to provide breast milk to their children but are unable to breastfeed; companies that manufacture products made out of breast milk for babies [19]; novel foods including breast milk cheese, ice cream and confectionery [20]; research [21] and other users, including older children, cancer patients [22], athletes [23] and some providers of sexual services [24]. To alleviate shortages, breast milk is shared locally and nationally through networks of milk banks [25-27] and less formally, via social networks and the internet [28-32] and rarely, internationally as overseas aid [33].

Concern about potential microbial and chemical contamination of breast milk has attracted the attention of regulators [34] while competition for surplus milk has fuelled debate about its allocation and systems of remuneration (donation or payment) [35,36]. Questions remain about the potential for exploitation [37-39] and the socioeconomic circumstances under which women breastfeed and produce milk [40].

Clear evidence of the value of breastfeeding to the economy of high income countries through prevention of health costs [41-43] and knowledge of what is required to improve breastfeeding rates [44,45] have failed to make breastfeeding a priority for policy makers in developed economies [42,46]. Breastfeeding rates are slow to change [2,45,46]. However in the near future, global population pressure on the supply of ingredients for breast milk substitutes will increase [47]. The interdependency of demand, supply and value (markets) of infant foods outlined above may exacerbate, rather than alleviate these problems and raise new challenges for human rights. A key question is whether existing strategies can increase rates of breastfeeding fast enough to meet future needs. In an era of rapid market liberalisation, free trade agreements and as breast milk is increasingly commodified, review of policies and national and international regulatory instruments to protect breastfeeding is required. These complex problems demand our urgent attention.

This article examines how the concept of food security applies to infants and young children, and identifies the key role of breastfeeding women as producers as well as providers of an available, appropriate, resilient source of food. It explores the potential of a food security framework to ‘recast the narrative’, focus disparate voices and foster the leadership required to address policy conflicts surrounding breastfeeding [48].

## Discussion

### Definition of food security

Concepts of food security have developed from a focus on the supply of food to its current definition, established in 1996 and confirmed in the 2009 Declaration of the World Summit on Food Security convened by the Food and Agriculture Organization of the United Nations: ‘Food security exists when all people, at all times, have physical, social and economic access to sufficient, safe and nutritious food, which meets their dietary needs and food preferences for an active and healthy life.’ ([49] p. 1). Although the importance of breastfeeding was recognised in the 1996 Rome Declaration on World Food Security [50], breastfeeding policy was developed within infant and maternal health and nutrition domains, and received limited attention in the wider ‘food security’ discourse [51-54].

Concepts of ‘food security’ and ‘nutrition security’ overlap [55]. Breastfeeding indicators are included in national and global nutrition-specific programs [56,57] and breastfeeding may be a goal or intervention within ‘nutrition security’, ‘food security’ and ‘global nutrition system’ frameworks [57-59]. In agriculture and other sectors, broader ‘nutrition-sensitive’ strategies are now called for to improve maternal and child health [60].

More recently, food security research has shifted emphasis from questions of supply to distribution and utilisation within food systems [59,61]. The common elements of food security in these approaches are food availability, accessibility (which includes affordability), utilization, and stability [62]. This paper applies these elements to the following infant foods: breast milk (through breastfeeding and expressed breast milk) and breast milk substitutes (infant formula, follow-up formula, toddler milks and unformulated animal milks) (Table 1).

**Table 1:** **Summary of components of food security for infants and young children and indicators**

**Food security term**	**Infant feeding system**		
	**Breastfeeding and wet-nursing** ^**1**^	**Expressed breast milk**	**Breast milk substitutes (BMS)**
Appropriateness	WHO/UNICEF Global Strategy for Infant and Young Child Feeding (2003) –breastfeeding most preferred of 4 options. Suitability of wet-nursing depends on circumstances. Health of mother/wet-nurse.	WHO/UNICEF Global Strategy for Infant and Young Child Feeding (2003) –suitability of milk from mother or donor depends on circumstances. Health of donor.	WHO/UNICEF Global Strategy for Infant and Young Child Feeding (2003) –suitability of BMS depends on circumstances. Compliance with composition, food safety and labelling standards for infant food^3^.
Availability	Rate of exclusive breastfeeding at 6 months. Continued breastfeeding for up to two years of age or beyond. Prevalence of wet nursing.	Location and support of breast milk banks and milk sharing. Transport and handling cold chain.	Retail outlets, including pharmacies, or rarely on prescription-only^4^. Local or imported brands.
		Feeding equipment and sterilization.	Clean water, feeding equipment and sterilization.
Accessibility	Economic and social policies that facilitate proximity between breastfeeding dyad: BFHI^2^; Maternity leave; Breastfeeding-friendly childcare; Breastfeeding-friendly work place. Acceptance of wet-nursing.	Donor and recipient must meet eligibility criteria for breast milk banks, milk sharing sites and social networks. Access to some milk banks may be on prescription only.	Retail outlets, including pharmacies, or on prescription-only^4^. Policies that separate mother and child. Regulation of marketing of BMS.
Affordability	Economic and social support for time, labour and opportunity cost to breastfeed. Financial, gift or social remuneration of wet-nurse.	Costs may include shipping, compensation or payment to milk bank or donor. Breast pump, feeding equipment and sterilization, storage and transport costs.	Requires financial payment. Retail cost of powdered or liquid infant formula varies with location and whether domestic or imported product used. Feeding equipment and sterilization costs.
Utilization	100% if milk production and intake regulated by breastfeeding dyad.	Wastage in handling, storage, distribution and feeding. Diversion from food chain.	Reduces consumption of breast milk. Wastage in BMS supply chain, manufacturing, distribution and feeding.
Stability	High rates of exclusive breastfeeding and proximity of breastfeeding dyad required. Stable in emergencies, unless relief disrupts breastfeeding. e.g. by supply of BMS.	Variation in supply not investigated. Expressing milk may increase supply of breast milk. Unstable in emergencies.	Feeding breast milk substitutes decreases supply of breast milk. Market access, including free trade agreements. Unstable supply and added risks in emergencies. May be oversupply of BMS as emergency relief.

1. Wet-nursing includes cross-nursing arrangements.

2. WHO/UNICEF Baby-friendly Hospital Initiative (BFHI).

3. Codex Alimentarius standards relevant to infant foods.

4. WHO International Code of Marketing of Breast-milk Substitutes (1981) and resolutions.

### Availability

Food availability is further described as ‘…sufficient quantities of food of appropriate quality, supplied through domestic production or imports (including food aid).’ ([62] p. 1). Breastfeeding is simultaneously the ‘domestic production’ and supply of food of appropriate quality for infants and young children, in accordance with WHO/UNICEF guidelines [1]. These guidelines stipulate both supply and appropriateness, as follows: ‘The vast majority of mothers can and should breastfeed… Only under exceptional circumstances can a mother’s milk be considered unsuitable for her infant.’ ([1] p. 10). Appropriateness of food also includes its social context, which for infants and their mothers, differs between breastfeeding, feeding of expressed breast milk and feeding breast milk substitutes.

In terms of sufficiency, the policies and practices required to support breastfeeding in different settings are known [2,45,46,48] but wherever breastfeeding rates are suboptimal, it follows that breastfeeding and breast milk are available inequitably. The total amount of breast milk available to a population is the amount consumed by breastfed babies plus any surplus, taking into account all sources (mothers, wet-nurses and expressed breast milk via milk banks and milk sharing). Estimates of breast milk production obtained from breastfeeding rates are included in national food production statistics in Norway [63], and have been used in studies of the volume and economic value of breast milk produced in Australia, the United States [42] and Sub-Saharan Africa [64]. However, these calculations do not include surplus breast milk.

The total amount of surplus breast milk and its effect on food security for infants at national levels are not known. Expressed breast milk can sometimes fill a short- or long-term gap in the supply of breast milk if a child has difficulties feeding at the breast or following maternal death, illness, absence or use of contraindicated medicines or drugs. Most current milk banking and sharing depends on breast pump use but the relationships between pump use and milk yield and breastfeeding rates and duration are unclear [65].

The variability in daily milk yield between women breastfeeding single babies has been studied [66] but less is understood about human potential yields. Measurement of maximum potential milk yield is complicated by homeostatic regulation, in which the volume produced is largely regulated by the amount removed [67]. The capacity of well-nourished women with sufficient time and support to breastfeed exclusively two or more rarely, three babies has been documented [68]. Similarly, milk bank data and media reports suggest that a proportion of mothers are able to produce a large surplus of milk using an electric pump, with records of up to 300 litres over 8 months [69], and extreme daily yields of 3.9 and 5 litres [70,71]. However, women often perceive their milk supply to be insufficient [12,72] and this lack of confidence contributes to premature weaning and is readily exploited in marketing strategies for breast milk substitutes [73].

The occurrence of milk banking is not necessarily associated with high rates of breastfeeding, although it is consistent with the WHO/UNICEF Baby-friendly Hospital Initiative and WHO/UNICEF Global Strategy on Infant and Young Child Feeding [74], and reduces the health risks and costs associated with feeding breast milk substitutes in neonatal intensive care units [75-77]. The volume of milk dispensed is disclosed by some milk banks [36,78,79] but information about the volume collected, discarded or diverted to other uses can be difficult to obtain [80]. As an indication of the amount of breast milk provided in the context of strong Government support, in 2011 in Brazil, over 200 milk banks accepted 165,000 litres of breast milk donated from 166,000 mothers which was distributed to nearly 170,000 babies [81]. In the United States in 2012 the 16 milk banks of the Human Milk Banking Association of North America (HMBANA) dispensed 74,000 litres [36], which represented a small proportion (about 0.01%) of the estimated 526 million litres consumed in the United States by breastfed babies in 2010 [42]. The added volume of breast milk distributed privately, or more publically via the internet, is difficult to quantify. A recent study of over 500 individuals donating milk on one website in the United States showed that nearly 50% of offers over a three-month period were for volumes of three litres or more [82].

The recent expansion of milk banking and sharing practices in different settings, especially in countries with low breastfeeding rates [27,36,82,83], are the subject of intense ethical debate [39], but less frequently considered in the context of infant food availability. Concerns that milk banking might reduce the motivation of mothers to provide their own milk has not been borne out in neonatal intensive care units [75,84-86]. Alternatively, milk banking and sharing could divert resources otherwise spent on improving breastfeeding rates, entrench poor breastfeeding policies, or replace breastfeeding for healthy full term babies.

The availability of breast milk substitutes is indicated by retail sales data for baby food and estimates of consumption by infants and young children of milk from cattle, buffalo, goat and other species. Sales of standard infant formula, follow-up formula, toddler milks and special baby formula in 80 countries amounted to 2.2 million tonnes, worth US$39.7 billion in 2013 [11]. These figures indicate how the current deficit in breastfeeding is being met. Further analysis of import statistics reveals the relative contribution of domestic and overseas supplies of breast milk substitutes and the length and complexity of globalized supply chains for infant foods [87]. Infants and young children are especially vulnerable to failures in the integrity and reliability of these supply chains. In contrast, the food availability and ‘supply chain’ for a breastfed infant is based on the proximity of the mother. It can be argued that food security for infants and young children in countries like China and Australia depends, ultimately, on the capacity to breastfeed.

### Accessibility

The accessibility of food is defined as ‘Access by individuals to adequate resources (entitlements) for acquiring appropriate foods for a nutritious diet. Entitlements are defined as the set of all commodity bundles over which a person can establish command given the legal, political, economic and social arrangements of the community in which they live (including traditional rights such as access to common resources).’ ([62] p. 1).

Access to breastfeeding requires access to a lactating woman –a mother, relative, friend or wet-nurse– and the infant’s ability to breastfeed. Separation of the breastfeeding dyad is entrenched in many policies and practices to do with post-natal care in hospitals, illness, maternity leave, work, childcare and sleep arrangements, and cultural and religious beliefs that influence breastfeeding in public and the age of cessation of breastfeeding [88-90].

Access to breast milk, is distinct from access to breastfeeding. The accessibility of expressed milk from a mother’s stored milk, milk banks, or social networks (including the internet), depends on appropriate equipment and transport. Breast pumps are common items in some high-income countries and research indicates that milk expression was practiced by over 70% of breastfeeding mothers of healthy full term babies in Australia and the United States [91,92]. Refrigeration and freezing extend the shelf life of breast milk [93]. Milk is usually pasteurised by milk banks, although milk that is not heat treated is used by milk banks in Norway [94] and is also shared informally elsewhere [29,32]. Alternative processing and packaging technologies are also being developed [95]. Systems for the collection and distribution of breast milk vary from mothers who transport their own expressed milk to delivery by firefighter and postal services in Brazil [96]; regional depots for milk banks in North America [26] and local delivery or postal services for milk sourced via the internet [29,32].

However, not all children qualify as recipients from milk banks, which typically prioritise premature or ill babies [26]. Donors of shared milk may also determine who obtains their milk (but have little control over its end use in practice). Donor criteria include the recipient’s need, relationship and other factors [82,83]. Access may also be determined by societal views that peer sharing of breast milk is an act of common humanity, a gift or a resource that should be shared rather than sold [28,39]. Alternatively, milk sharing may be considered an unacceptable activity that is disgusting or perverted [97]. In contrast, concepts of ‘milk kinship’ in Islamic cultures may support milk sharing but restrict access [98]. Detailed analysis of the distribution of expressed breast milk in a population has not been undertaken [82].

The complex factors that influence food distribution and access are recognised in concepts of food security. Use of milk sharing and wet-nursing allows babies to access milk from women who are better able to breastfeed by virtue of the ‘legal, political, economic and social arrangements of the communities in which they live’ ([62] p. 1). If a woman is unable to breastfeed because of insufficient support from her health system, family, workplace, child care or community, she can theoretically source breast milk from a woman who is so supported. Concepts of food security acknowledge that social inequity, and cultural and institutional barriers determine access to breastfeeding and breast milk. In contrast, popular discourses often attribute decisions to breastfeed to individual choice or agency alone [89,99].

Access to breast milk substitutes is determined by the distribution of retail outlets in most countries for other highly processed foods, pharmacies, and, less commonly, on prescription. In countries where the WHO Code [16] and resolutions [14,18] are not observed, direct supply to mothers occurs through hospitals [12,100], health professionals [101,102], the internet and government welfare programs, for example the Special Supplemental Nutrition Program for Women, Infants and Children (WIC) in the United States [103]. The safe reconstitution and feeding of breast milk substitutes also requires skill and access to supplies of energy and potable water.

### Affordability

Affordability is a key component of access to food that depends on a household’s economic circumstances. Countries and states vary in their regulation of donation and payment for breast milk, reflecting prevailing societal values [104-106]. However, breastfeeding provides much more than the production and transfer of food, and includes less tangible associations with mental and emotional health of the child and mother [107,108].

Donation-only models for breast milk are based on principles of gifting human blood and tissues [109] and raise ethical challenges of supply, cost and risk management [110] but may not fully account for breast milk as a food. Concerns about exploitation are similar to those applied to wet-nursing, namely that women might produce and sell milk against their own best interests or those of their child. These concerns need to be viewed in the light of societal expectations that mothers breastfeed without recognition of or recompense for the time, labour and opportunity costs of doing so [111].

Currently, access to banked breast milk requires payment to a milk bank or hospital and its affordability for families depends on cost sharing arrangements between health systems and insurers [36]. Payment is not accepted by most peer milk sharing networks and compensation (for example, providing containers for milk) is discretionary. Payment of donor mothers by the first milk banks in the United States in 1909 and the United Kingdom (from 1939 until 1985) was necessary to secure supplies of milk and followed the historical practice of payment of wet-nurses [112]. With the resurgence of non-profit milk banking in the latter part of the 20^th^ century, donors were not paid but questions about the supply and affordability of human milk remain [37]. Currently, milk provided by milk banks in the United States and Norway costs US$101-$228 per litre to cover the costs of screening donors and milk testing and processing [42,113], while milk banks in Denmark pay donors and do not charge recipients [104]. Information on prices for wet-nursing services is limited [32,42].

The cost of expressed breast milk obtained from unregulated sources is often prohibitive for households unless it is donated. Health assessment and testing of donors may be arranged by recipients, depending on their knowledge, perceptions of risk and the level of relationship or trust established with the donor [114]. Prices (excluding shipping) for expressed breast milk sold online of US$34-$101 per litre (US$1-$3 per oz.) in the United States appear stable over recent years, while in the United Kingdom breast milk is more expensive (US$112-$170 per litre), possibly reflecting the smaller size of the market [32,42,95]. The cost to hygienically prepare feeds, including that of access to clean water, must be added to the costs of both expressed breast milk and breast milk substitutes.

In contrast, the price of breast milk substitutes for babies 0–12 months old in the United States was US$1.50-US$7.00 per litre for reconstituted powder and US$7-$27 per litre for ready to use (liquid) infant formula. Prices increased to US$20-$35 per litre for products labelled as ‘organic’ or for ‘special needs’ and US$104 per litre for products labelled ‘hypoallergenic’ [115]. In China, foreign-owned brands comprise about half the market for infant formula, with prices two to three times higher than in the United States, Europe and Australia [87].

Breastfeeding may be unaffordable if the household cannot afford to lose the mother’s income, or the mother cannot provide the time and labour to breastfeed [111], express milk, or obtain adequate workplace and child care support to do so [12,116,117]. Maternity protection and anti-discrimination legislation, where these are implemented, may not effectively mitigate these costs or the loss of the mother’s future earning capacity [118]. An earlier return to the paid workforce after the birth of a child is associated with a shorter duration of breastfeeding [119,120]. Irrespective of the cause of premature weaning, its occurrence requires households to accommodate the cost of breast milk substitutes and associated short- and long-term health costs to the child and mother.

The affordability of breastfeeding also includes the cost to workplaces of supporting breastfeeding employees by providing facilities, time and flexible work arrangements [119,121]. Distribution of this cost between employers, employees and wider society, reflects the extent to which breastfeeding is normalised and protected in that society or remains a source of gender inequity. Tools are available to assess the national costs of implementing effective measures to protect, promote and support breastfeeding [122,123].

### Utilization

The definition of food security includes ‘Utilization of food through adequate diet, clean water, sanitation and health care to reach a state of nutritional well-being where all physiological needs are met. This brings out the importance of non-food inputs in food security’ ([62] p. 1). Breast milk fed by breastfeeding or wet nursing meets all the criteria of utilization. Utilization of expressed milk is more contentious. Expressed breast milk that fails to meet safety and quality standards for milk banks and milk sharing recipients may be lost to the food chain. Screening of donors and testing and pasteurisation of milk reduce some of this wastage [36]. A proportion of expressed breast milk is also lost during collection, storage and feeding.

Utilization of breast milk raises potential human rights issues. Lactating women may have limited control over the end use of their milk, for example through a lack of transparency by milks banks or other recipients of donated milk or requirements in marital, divorce or surrogacy arrangements to supply breast milk [124-126]. There is limited understanding of how much breast milk women would produce and make available for different users for altruistic or financial reasons and if protected from exploitation. Studies of not-for-profit (donation-only) milk sharing sites and breast milk banks show that most of their donors are strongly motivated by altruism [127,128]. However, in locations where it is possible to choose between selling and donating breast milk, the amounts sold and donated have not been compared. Financial incentives are likely to appeal most to unemployed or poor women, without adequate support or maternity protection. Recently, shopping vouchers were offered to disadvantaged women who breastfeed in the United Kingdom [129], and limited access to maternity leave was a rationale for payment by a breast milk cooperative in the United States [95]. These strategies, and the debates surrounding them, are unique to the cultural, legal, political and economic settings in which they arise [37,38,130].

The efficiency of breastfeeding compared with other methods of feeding infants is of fundamental importance to food security. Utilization includes concepts of efficiency of resource use from processes of production through to consumption. Wastage from artificial feeding occurs through the use of land, fertilizer, water, energy and materials to make and use breast milk substitutes, teats and bottles. The environmental impacts of these processes include pollution of natural resources, transport and disposal of wastes, effluent and packaging [131,132], which are also relevant, in part, to storing and feeding expressed breast milk. Life cycle analyses show that the dairy sector consumes large amounts of water [133] and contributes about 2.7% of global anthropogenic greenhouse gas emissions [134], most of which occur on farms. Research on the stability of global agricultural production and food supply under a range of resource-limited scenarios needs to be linked to policies for infant and young child feeding.

### Stability of supply

The vulnerability and growth needs of infants and young children give them little capacity to tolerate unstable supplies of food. ‘To be food secure, a population, household or individual must have access to adequate food at all times. They should not risk losing access to food as a consequence of sudden shocks (e.g. an economic or climatic crisis) or cyclical events (e.g. seasonal food insecurity). The concept of stability can therefore refer to both the availability and access dimensions of food security.’ ([62] p. 1).

A household’s supply of breast milk is not stable unless a woman is able, motivated and supported to breastfeed, or she can access milk from another lactating woman. Separation of the mother from her child may be managed if distance and time permit access to breastfeed, or carers use stored breast milk. Outside the regulated childcare sector, some mothers arrange for their child to be cross-nursed.

Temporal and geographic variation in supply to breast milk banks and milk sharing sites are not well understood [82] and may include fluctuations in the time available to express milk, awareness of milk banking and sharing, skill in expressing milk, factors that influence weaning, the donor’s use of medicines or herbal preparations, the age of her baby and guidelines on milk storage [26,135]. The breast milk supply chain to milk banks and sharing networks depends on constant recruitment of donors from a population with adequate breastfeeding rates. Shortages of milk are reported frequently by milk banks [36]. In the first years after human immunodeficiency virus appeared, recommendations for infected women to use breast milk substitutes or pasteurise their milk [136] reduced breastfeeding rates in some countries and closed milk banks in others [137].

The supply of breast milk substitutes can be destabilised by disruptions to manufacturing processes and supply pipelines, distribution and marketing systems, food safety recalls and changes to food regulations and corporate decisions. Instability in a household’s supply of breast milk substitutes may occur because of their high cost relative to income or economic shocks, for example sudden unemployment.

Emergencies, natural disasters and civil unrest challenge all aspects of food security. A key responsibility of international aid agencies and governments is to plan for infant feeding in these situations [138,139]. Breastfed infants and young children are food secure if their mother or another lactating woman is accessible and well enough [140], with little additional food, shelter and social support [141]. In emergency situations the food security of artificially fed infants and young children can change abruptly due to disruption to the supply and affordability of breast milk substitutes [142] or a lack of resources and equipment to feed powdered infant formula safely [143]. Guidelines have been developed for international aid agencies to support breastfeeding mothers in emergencies and prevent the donation and distribution of breast milk substitutes that destabilize breastfeeding practices [139,143]. However these guidelines were not observed in the aftermath of earthquakes in the Asia Pacific region (Yogyakarta and Central Java in 2006, Sichuan Province in 2008 and the north-east Japan earthquake and tsunami in 2009) [142], Cyclone Nargis in Myanmar in 2008 [144] and the Haiti earthquake in 2010 when large amounts of infant formula were distributed despite the use of baby tents to maintain breastfeeding in the latter [145,146]. Outbreaks of Ebola virus in West Africa in 2014 present new challenges to breastfeeding [147].

## Responses to food insecurity for infants and young children

Food insecurity can be considered a form of ‘market failure’, in which the production or exchange of food is inadequate [148]. When applied to infant feeding, responses to this failure include marketing, regulation or a combined approach, including self-regulation through private standards and private-public partnerships [148,149].

### Trade

The conventional response to food insecurity is trade. Imports can contribute to food security when local production cannot meet demand. The contribution of wet-nursing services and expressed breast milk to meet a ‘supply gap’ [34] may enhance infant food security in the short term, while their long term effects on breastfeeding capacity at individual and population levels are less clear.

The question of whether trade in substitutes for breast milk improves food security depends, in part, on their effect on breast milk production. Data from 2002–2013 show that annual milk formula sales increased rapidly in China but remained much lower in India [11,48], while rates of exclusive breastfeeding were lower in China (28%) than India (46%) for children aged 0–5 months in 2006–2010 [13]. By 2013 in China the retail value of milk formula for infants and young children aged 0–36 months reached US$16.0 billion compared with US$0.5 billion in India [11]. These outcomes reflect India’s greater regulation of marketing of breast milk substitutes, as well as differences between these countries in their economic environments and implementation of the WHO/UNICEF Global Strategy for Infant and Young Child Feeding [45,48].

Free trade agreements may affect infant food security if they change access to and reduce prices of breast milk substitutes or undermine health policies. Of concern are Investor-State Dispute Settlement (ISDS) clauses in trade agreements that may allow legal action to be brought against a government if the trade environment changes [150]. Negotiation is currently underway of the Trans-Pacific Partnership (TPP) Agreement between Australia, New Zealand, the United States, Peru, Chile, Mexico, Canada, Singapore, Brunei, Malaysia, Vietnam and Japan. This agreement has the potential to account for 37% of total global gross domestic product (GDP) [151] without China, which is expected to join in the future. While the details of the TPP are not known, it may create a much larger and less regulated market in which public health advocates attempt to protect breastfeeding. Arguably, this could occur if public health policies were introduced that restricted marketing of or access to breast milk substitutes. The potential effect of these agreements on infant food security has not been investigated.

### Policy

Appropriate policy responses to infant food security require recognition of the conflicting objectives of the public and private sectors and consultation with a broad range of stakeholders. Increasingly, partnerships between government and industry are proposed as solutions to food security issues [152]. However their adoption for infant and young child food security requires protection of breastfeeding including through stringent observance of the WHO Code [16,59,153]. At a global level, high rates of breastfeeding are a cornerstone of infant and maternal nutrition programs to achieve Millennium Development Goals (MDGs) which aim to reduce stunting (MDG 1), child mortality (MDG 4) and improve maternal health (MDG 5) [154]. It is argued that coordination between governments and international agencies is necessary to improve progress in these MDG targets, for example through the ‘1,000 Days’ [56,155] and Scaling Up Nutrition (SUN) [57] initiatives. However breastfeeding outcomes may be undermined if policies in donor countries and non-governmental organizations are dominated by ‘product and market based approaches’ to aid ([48] p. 26) or trade opportunities, for example the export of dairy-based breast milk substitutes [132,156]. These policy conflicts may entrench poor infant health outcomes worldwide and inhibit the policy development required to improve breastfeeding rates and long-term food security in both exporting and importing countries [54,157].

### Regulation

Food regulation attempts to address deficits in the quality, safety and marketing of food. National food regulations typically refer to international standards of the Codex Alimentarius [158], in which manufactured infant foods have the most extensive standards of any food category [159]. Consistent standards of composition and labelling also facilitate trade. Regulatory approaches to infant feeding that are linked to broader concepts of food security warrant more detailed analysis than can be undertaken here but some emerging issues are outlined below.

Breast milk expressed by a mother for use by her own child is largely unregulated, although guidelines and health recommendations to store and handle breast milk safely may be part of workplace and childcare centre policies [117,160]. A range of regulatory issues surround breast milk provided for other users, depending on its classification as a food or human tissue [75,106], the legality of its donation or sale and whether it is controlled by milk banks or hospitals or shared in the community and via the internet. Milk banks use a range of quality and safety guidelines and manufacturing standards [26,93,135]. Concerns over the safety of shared milk obtained from unregulated sources have prompted calls for quality standards and regulation of this trade [34]. For those infants whose mothers are unable to fully breastfeed them and are not eligible for banked donor milk, the challenges for health authorities and consumers are to assess and manage the risks of shared milk relative to breast milk substitutes, in terms of food safety and short- and long-term infant and maternal health [28]. The effects of any regulatory measures on the affordability and access to shared breast milk [97] are also food security issues. Contamination of breast milk with drugs, medicines or chemical residues present in the mother’s environment depends on their clinical significance and applies to breastfed children generally, as well as those receiving shared milk [161,162]. Low-cost methods of labelling, packaging and storage are important for the safe use and affordability of breast milk [93] and international standards for identification of breast milk have been proposed [163].

It should be noted that breast milk substitutes are not sterile products and are therefore subject to the risk of microbiological, as well as chemical and foreign body contamination [164,165] and reconstitution with contaminated water [166]. Recent efforts by the Chinese government to re-establish consumer confidence in breast milk substitutes manufactured in China include industry consolidation [87,167] and more stringent food safety standards [8,168]. These changes may decrease demand for imports into China and stabilise the availability of breast milk substitutes in other countries.

The adequacy of food standards for breast milk substitutes and their regulatory oversight raise important food security issues [159,168-170]. International and national food standards and regulations must also be kept up to date with WHO Code and relevant WHA resolutions [14,159,171], as misalignment can be used as a basis for legal action against regulation of marketing [17,172]. Implementation of the WHO Code is also challenged by the marketing of breast milk substitutes via the internet and social media [173]. Non-regulatory measures, such as boycotts and publicity about the marketing behaviour and corporate ethics of infant food companies, have been used for several decades [174].

Regulatory approaches to food security also intersect with human rights principles and cultural and religious beliefs [175]. Resolution of these conflicts may require the adoption of perspectives that focus on empowerment [54,176] or recognise the rights of the mother-child dyad as a unit rather than individuals or conjointly [39]. Islamic principles that children should be breastfed for two years and systems of ‘milk kinship’ require recognition in hospital, milk banking and milk sharing practices [98]. Laws to uphold a child’s right to be breastfed were introduced in Indonesia in 2009 [177] and the United Arab Emirates in 2014 [126]. These laws apportioned responsibility differently. The Indonesian law stipulated penalties for those who prevented exclusive breastfeeding for six months [178], while the Emirati law was reported to emphasise the individual mother’s responsibility to breastfeed for two years [126].

### Future responses to food insecurity of infants and young children

By 2050, within one generation interval, the world will need to feed a predicted population of 9.6 billion people [179]. In this scenario, infants and young children fed breast milk substitutes will be especially vulnerable to increased global competition for high protein dairy and soy products [59,168,180]. Food price spikes and political instability that arise when food becomes unaffordable are crisis situations that are unlikely to prompt resolution of complex and long-term problems within a whole food system. For example, the Chinese government responded to price increases for imported breast milk substitutes by tightening food regulations but it is less clear what measures were taken to improve breastfeeding rates.

## Conclusions

Food security for infants and young children is not yet perceived as a major problem in most high-income countries. However, studies of the short- and long-term health risks of inadequate breastfeeding indicate that breast milk substitutes fail to meet the objectives of the Rome Declaration on World Food Security as ‘sufficient, safe and nutritious food that meets …dietary needs and food preferences for an active and healthy life’ ([50] p. 3). A high rate of breastfeeding is a marker of the cultural appropriateness and utilisation of food for infants and young children, as well as its supply. Yet without the social, legal and economic rights that protect breastfeeding, infants do not have a secure supply of food.

This paper proposes that low rates of breastfeeding in many countries may be improved by adopting food security approaches. Food security for infants and young children emphasises the relationships between the supply, availability and affordability of all infant foods and recognises the work of breastfeeding women as food producers. A food security framework includes food utilization and efficiency that will be critical for the world’s future capacity to feed infants and young children optimally. New ways of conceiving systems of governance are required to manage the emerging challenges from resource limitations and a less-regulated, globalized trade environment for infant foods.

The application of food security concepts to infant and young child feeding may foster a sense of the urgency, political will and the broader frameworks required to review, coordinate and implement effective infant feeding policies.

### Summary

The environment in which mothers and governments make decisions that affect infant and young child feeding is changing rapidly. Inadequate progress in breastfeeding rates worldwide over the past few decades and new patterns of globalised trade facilitated by the internet, challenge existing national and international health policies intended to protect, promote and support breastfeeding. Low rates of exclusive breastfeeding and recent ‘shortages’ of supplies of expressed breast milk and breast milk substitutes in low-, middle- and high-income countries highlight the food insecurity of infants and young children.

This paper proposes that concepts of food security –food appropriateness, availability, accessibility, affordability, utilization and stability of supply– apply to infants and young children and that high rates of optimal breastfeeding are required for this group to be food secure.

Food security provides an analytical framework and overarching policy imperative that may help international agencies, governments and community organizations to better address conflicts between health, agriculture and trade, all of which contribute to low breastfeeding rates and unregulated trade and marketing of breast milk substitutes. Existing policies fail to account for human rights and the unpaid work of breastfeeding women.

Breastfeeding improves global capacity to adapt to future food security challenges arising from predicted global population expansion, shifts in climate and limits to agricultural and industrial production of infant foods. Applying concepts of food security to infants and young children might foster the political will, policy coordination and economic changes required to improve breastfeeding rates.

## Abbreviations

BMS: Breast milk substitutes
HMBANA: Human Milk Banking Association of North America
MDG: Millennium Development Goal
UNICEF: United Nations Children’s Fund
WHO: World Health Organization
